# A PTK7/Ror2 Co-Receptor Complex Affects *Xenopus* Neural Crest Migration

**DOI:** 10.1371/journal.pone.0145169

**Published:** 2015-12-17

**Authors:** Martina Podleschny, Anita Grund, Hanna Berger, Erik Rollwitz, Annette Borchers

**Affiliations:** Faculty of Biology, Molecular Embryology, Philipps-Universität Marburg, 35043 Marburg, Germany; CHU Sainte Justine and University of Montreal, CANADA

## Abstract

Neural crest cells are a highly migratory pluripotent cell population that generates a wide array of different cell types and failure in their migration can result in severe birth defects and malformation syndromes. Neural crest migration is controlled by various means including chemotaxis, repellent guidance cues and cell-cell interaction. Non-canonical Wnt PCP (planar cell polarity) signaling has previously been shown to control cell-contact mediated neural crest cell guidance. PTK7 (protein tyrosine kinase 7) is a transmembrane pseudokinase and a known regulator of Wnt/PCP signaling, which is expressed in *Xenopus* neural crest cells and required for their migration. PTK7 functions as a Wnt co-receptor; however, it remains unclear by which means PTK7 affects neural crest migration. Expressing fluorescently labeled proteins in *Xenopus* neural crest cells we find that PTK7 co-localizes with the Ror2 Wnt-receptor. Further, co-immunoprecipitation experiments demonstrate that PTK7 interacts with Ror2. The PTK7/Ror2 interaction is likely relevant for neural crest migration, because Ror2 expression can rescue the PTK7 loss of function migration defect. Live cell imaging of explanted neural crest cells shows that PTK7 loss of function affects the formation of cell protrusions as well as cell motility. Co-expression of Ror2 can rescue these defects. *In vivo* analysis demonstrates that a kinase dead Ror2 mutant cannot rescue PTK7 loss of function. Thus, our data suggest that Ror2 can substitute for PTK7 and that the signaling function of its kinase domain is required for this effect.

## Introduction

Neural crest (NC) cells are a highly migratory pluripotent cell population that gives rise to a wide range of derivatives contributing to many tissues and organs. NC cells develop along the anterior-posterior axis of the vertebrate embryo at the border region between the epidermis and the neural plate. After undergoing an epithelial to mesenchymal transition NC cells migrate and colonize almost all tissues of the embryo. In this respect NC migration is very similar to cancer cell invasion and metastasis dissemination, which is also mirrored by the conservation of molecules and signaling pathways involved in both processes [1,2,3]. During migration NC cells follow precise pathways and encounter various molecular microenvironments, which guide them to their final destinations and determine their terminal differentiation [4]. Therefore, NC migration needs to be tightly controlled to ensure the development of multiple organs and tissues. Indeed, failure results in severe birth defects and malformation syndromes–so called neurocristopathies [5,6,7]. Thus, understanding the molecular mechanism that control NC migration will also provide insight into pathological conditions like neurocristopathies or the development of cancer.

Non-canonical– ß-catenin-independent–Wnt signaling contributes significantly to the regulation of NC migration [8,9,10,11,12,13,14]. NC cells show collective cell migration and form streams of migrating cells directed for example by repellent guidance cues and chemo-attractants [2,4]. In addition, NC cell directionality is achieved by communication of NC cells with each other. One mechanism, which was proposed for the directional migration of cranial NC cells is contact inhibition of locomotion. This phenomenon was discovered by Abercrombie [15,16] and describes how two colliding cells change their cell polarization upon cell-cell-contact and migrate in opposite directions. Recent work showed that non-canonical planar cell polarity (PCP) Wnt signaling is required for contact inhibition of locomotion and directional collective NC migration [8,9,10,17,18]. PCP signaling was first discovered in the fly where it regulates the orientation of hairs in the wing or ommatidia organization in the eye [19,20]. In vertebrates PCP signaling is necessary for inner ear patterning, ciliary beating and tissue movements contributing to axis elongation and neural tube closure [21,22]. In all these biological systems PCP proteins become asymmetrically localized to the plasma membrane thereby establishing a polarity in the plane of an epithelium. In moving tissues the situation is more complicated. In migrating NC cells PCP signaling seems to determine the formation of cellular protrusions by asymmetrically regulating the activity of small GTPases of the Rho family [14,18,23]. PCP proteins including Frizzled and Dishevelled are localized to the site of cell contact thereby leading to a local activation of Rho and inhibition of Rac [8,9,17,24]. Thus, cell protrusions collapse and cells migrate in opposite directions. Thereby, PCP signaling provides a means of NC cell dispersion, which in combination with other repellent, attractant and adhesive cues contributes to controlled migration of NC cells [23,25,26,27]. Although, the contribution of PCP signaling to NC migration has been acknowledged in different vertebrate systems the molecular components localizing to cell-contact sites and the signaling pathways leading to cytoskeletal remodeling remain to be characterized.

Protein tyrosine kinase 7 (PTK7, also known as Colon Carcinoma Kinase-4, CCK-4) a known regulator of planar cell polarity may be one of the players involved in cell-contact-mediated NC cell guidance. PTK7 is an evolutionary conserved transmembrane protein with extracellular immunoglobulin domains and an intracellular kinase homology domain, which lacks catalytic activity [28,29]. In *Xenopus* PTK7 is required for NC migration [13]. As PTK7 interacts with Frizzled7 and recruits Dishevelled to the plasma membrane [13,30], it could play a role in cell-contact-mediated NC cell guidance. Recent publications suggest that PTK7 affects multiple Wnt signaling pathways. Loss of function analysis in mice, zebrafish and *Xenopus* show that PTK7 is required for non-canonical Wnt signaling controlling convergent extension cell movements and planar cell polarity [31,32,33,34]. In addition, PTK7 function has been implicated in canonical Wnt signaling. Recently, we and others demonstrated that PTK7 interacts with canonical Wnt proteins [35,36] and inhibits canonical Wnt signaling [34,35]. Contradictory to these findings a role of PTK7 as an active component of canonical Wnt signaling has also been reported [37,38] and PTK7 was shown to interact with LRP6 [38]. Therefore, differences in the composition of the PTK7 co-receptor complex may explain these contradictory findings in different tissue settings. This is also supported by reports concerning PTK7 downstream signaling, where various signaling outcomes like activation of c-jun, AKT, Ras/ERK, CREB/ATF1, SRC and inhibition of JNK signaling were observed [39,40,41,42]. The fact that PTK7 interacts with other receptors like Plexin and VEGF receptors further supports this hypothesis and suggests that PTK7 is a versatile co-receptor [43]. Similarly, receptor context may also modulate PTK7 function in Wnt signaling, however, so far interaction with non-canonical Wnt receptors such as Ror2 have not been analyzed.

The receptor-tyrosine kinase Ror2 is an evolutionary conserved Wnt receptor that has been shown to activate ß-catenin-independent Wnt pathways and to antagonize Wnt/ß-catenin signaling [44,45,46,47,48,49,50]. The extracellular part of the Ror2 protein contains one Ig-domain, the Wnt-binding CRD domain, and one kringle domain; its intracellular part consists of a supposedly active tyrosine-kinase domain and a C-terminal serine/threonine- and proline-rich domain. The latter is the major interface for cytoplasmic interactions e.g. with Dishevelled, Filamin A, CK1 and GSK3ß [51,52,53,54]. In vertebrates, Ror2 was found to form a receptor-complex with Frizzled and is one major co-receptor in Wnt/Frizzled mediated planar cell polarity signaling [55,56] and Wnt-regulated cell movements [52,57,58,59]. In addition, Ror2 can act as a bona fide receptor tyrosine kinase to activate a PI3K-JNK cascade in gastrulating *Xenopus* embryos [44]. Ror2 is expressed in the dorsal mesoderm, the neuroectoderm, in pre-migratory and migrating NC cells in *Xenopus* embryos and in the cranial ganglia of tadpoles [57,60]. Thus, Ror2 and PTK7 are co-expressed in migrating NC cells and share a common function in the regulation of canonical and non-canonical Wnt signaling pathways suggesting that they may interact in the regulation of NC migration. Here, we provide evidence supporting this hypothesis. We find that PTK7 and Ror2 co-localize in NC cells and that they interact biochemically. Further, Ror2 can rescue the PTK7 loss of function NC migration defect indicating that it can substitute for PTK7.

## Materials and Methods

### Plasmid cloning

For cloning of the HA-tagged full-length mouse Ror2 (Ror2-HA) the coding sequence of mouse *ror2* was amplified using the following primers: forward *5’*ACGCTCGAGGTGCATCGGGGCAGGAAAGGGGAC3’ and reverse 5’ACGCTCGAGGGCTTCAAGCTGGACATGAGCCG3’. The PCR product was introduced into the XhoI restriction site of the pCS2+/HA vector. For myc-tagged full-length human PTK7 (PTK7-MT), the coding sequence of human *ptk7* was amplified using the following primers: forward 5’CACGTGATCGATGCCCTCAGCTCCTTTTCCTGA3’ and reverse 5’GACGTGATCGATGCGGCTTGCTGTCCACGGT3’. The PCR product was introduced into the ClaI restriction site of pCS2+/MT. Myc-tagged human PTK7 lacking the intracellular kinase homology domain (h ΔkPTK7-MT) was amplified from PTK7-MT using the following primers: forward 5’CACGTGATCGATGCCCTCAGCTCCTTTTCCTGA3’ and reverse 5’CCGTATCGATCGGGCTCCTTCTGCAGC3’. The PCR product was introduced into the ClaI restriction site of pCS2+/MT. For the secreted extracellular domain of mouse Ror2 (sRor2-HA) the extra cellular part was amplified using the following primers: forward 5’CAGCTCGAGCCGCAGCATGGCTCGGGGCTGG3’ and reverse 5’gctgCTCGAG acacgggggtacgtcacacagttg3’. The PCR product was introduced into the XhoI site of pCS2+/HA. The *Xenopus* RFP-tagged PTK7 (PTK7-RFP) was cloned using a 3 step PCR with a pair of chimeric primers thereby fusing the RFP-tag in frame to the C-terminus of PTK7.

### *Xenopus* injection

*Xenopus* embryos were generated and cultured according to general protocols and staged according to the normal table of Nieuwkoop and Faber [61]. All procedures were performed according to the German animal use and care law (Tierschutzgesetz) and approved by the German state administration Hesse (Regierungspräsidium Giessen). For *Xenopus* microinjection capped sense mRNA was synthesized using the mMessage Machine Kit (Ambion, Life Technologies). The following published plasmids were used: LacZ [62], mGFP [63], H2B-mcherry [64], *Xenopus* Ror2-3I [57], mouse Ror2∆469-Flag [65] and *Xenopus* Ror2-EGFP [60].

For the PTK7 loss of function rescue experiments different combinations of MO and sense mRNA were co-injected. 10 ng of PTK7 MO (a combination of two different MO was used as previously described [30]) or standard control MO (GeneTools) were co-injected with 100 pg *mGFP*, mouse *Ror2*, mouse *Ror2Δ469* or *Xenopus Ror2-3I* in one blastomere of a two-cell stage embryo. In addition 100 pg *LacZ* RNA was injected as a lineage tracer. Cultivation, staging and fixation of embryos were performed as previously described [66]. Embryos were cultured until tadpole stages (stage 26–28) and then further analyzed using ß-galactosidase staining and *in situ* hybridization using the NC marker *twist*.

### Cranial NC explants and time-lapse imaging

For NC cell explantation embryos were microinjected with 50 pg *mGFP* mRNA and 250 pg *H2B-mcherry* mRNA together with 7.5 ng control MO or PTK7 MO in one blastomere at the two-cell stage; for rescue experiments 150 pg *Ror2* RNA were co-injected. To analyze for co-localization of PTK7 and Ror2 embryos were injected with 400 pg PTK7-RFP and 150 pg Ror2-EGFP. Explantation of NC cells was performed as described [67]. Explanted NC cells were placed in fibronectin coated (1 mg/ml in PBS) chamber slides (Lab-Tek Chambered Coverglass (Thermo Scientific)) filled with DFA medium (53 mM NaCl, 5 mM Na_2_CO_3_, 4.5 mM K-Gluconacid, 32 mM Na-Gluconacid, 1 mM MgSO_4_, 1 mM CaCl_2_, 0.1% BSA, adjusted with bicine (1M) to pH 8.3). Explants were dissected into smaller pieces using an eyebrow-knife and incubated for two to four hours at 14°C to ensure adherence of the cells. Subsequently NC migration was observed and analyzed by spinning disk microscopy (AxioObserver Z1, Zeiss, Objective 10x plan apochromat NA 0,45 or 63x plan apochromat NA 1.4 oil). Images were taken every 30 seconds (for imaging at 63x) or every 60 seconds (for imaging at 10x) for a time interval of up to 8 hours.

Image analysis was performed using ImageJ. For cell tracking the MTrackJ plugin [68] was used. Briefly, for each condition 5 cells were tracked using the nuclear H2B fluorescence to follow their movement throughout all frames of the movie. Further, to analyze the dispersion of NC cells Delaunay triangulation was used. In short, the nuclei were selected and the Delaunay/Voronoi diagram plugin was used to draw a Delaunay diagram from the point selections.

### Co-immunoprecipitation and Western blot

For co-immunoprecipitation experiments MCF7 cells were transfected using Lipofectamine 2000™ (Life Technologies) with the following plasmids: Ror2ΔCRD/KR∆745-Flag, Ror2Δ745-Flag and Ror2∆469-Flag [65], Ror2-HA, sRor2-HA, PTK7-MT and ∆kPTK7-MT. In case of single plasmid transfection 2 μg of pCS2+ vector without insert were added to equalize plasmid concentrations. 48 hours after transfection cells were washed in TBS (50 mM Tris (pH 7.5), 150 mM NaCl), scraped and lysed in lysis buffer (TBS containing 0.5% NP-40 and Complete protease inhibitor mix EDTA-free (Roche)). 750 μl of total lysate were pre-cleared by continuous mixing with Protein A Sepharose CL-4B (GE Healthcare) for one hour at 4°C. After centrifugation 50 μl of the pre-cleared lysate were taken as input control. For co-immunoprecipitation mouse anti-HA (HA.11 16B12 Covance, dilution 1:150), mouse anti-myc (9E10 M5546 Sigma, dilution 1:500), goat anti-myc (ab 19234 Abcam, dilution 1:250) or mouse anti-Flag M2 antibodies (F4049 Sigma, dilution 1:500) were added to the supernatants and incubated at 4°C for two hours. Subsequently Protein A Sepharose slurry was added and probes were further incubated at 4°C for one hour. The Protein A Sepharose beads were washed five times with lysis buffer for five minutes at 4°C. The beads were mixed with 6x Laemmli loading buffer (0.35 M Tris (pH 6.8), 30% Glycerin, 10% SDS, 9.3% Dithiothreitol, 0.02% bromphenol blue), denatured at 95°C and loaded on 10% SDS PAGE gels. Detection of proteins by immunoblotting was carried out using different antibodies: goat anti-myc (ab 19234 Abcam, dilution 1:1000), mouse anti-myc (9E10 M5546 Sigma, dilution 1:2000), mouse anti-HA (HA.11 16B12 Covance, dilution 1:1000), goat anti-Ror2 (AF 2064 R&D Systems, dilution 1:1000). As secondary antibodies anti-mouse HRP (sc2005 Santa Cruz, dilution 1:5000) or anti-goat HRP (sc2020 Santa Cruz, dilution 1:10000) were used.

## Results

### PTK7 and Ror2 co-localize in NC cells and interact in co-immunoprecipitation experiments

**Fig 1:**
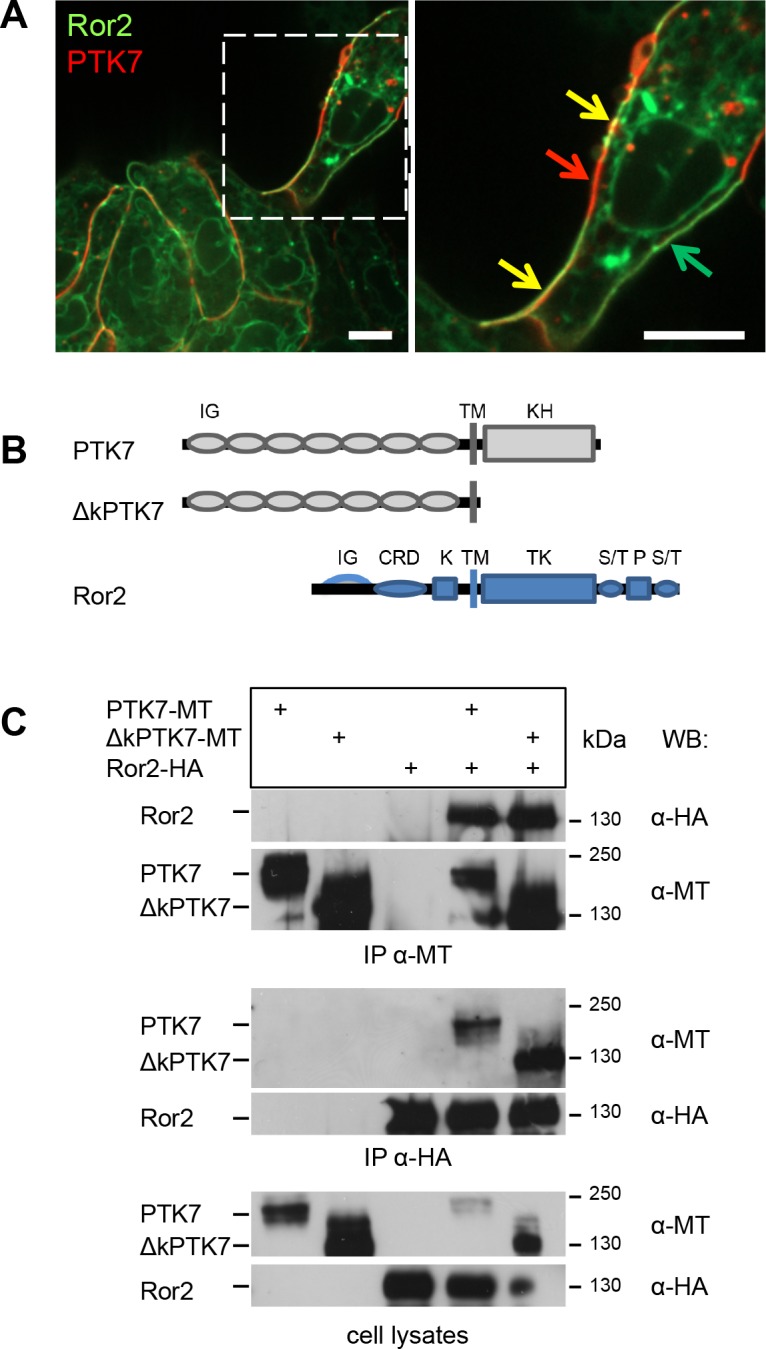
PTK7 and Ror2 co-localize in NC cells and co-precipitate independent of the kinase-homology domain of PTK7. **A** Co-localization of PTK7 and Ror2. NC cells co-expressing Ror2-EGFP and PTK7-RFP show distinct areas of co-localization (yellow) as well as membrane areas where only PTK7-RFP (red) or Ror2-EGFP (green) is localized. The right panel shows a higher magnification of the single cell in the left panel (indicated by a dashed square), scale bar = 10 μm. **B,C** Co-immunoprecipitation of PTK7 and Ror2. Full-length myc-tagged PTK7 (PTK7-MT) and a myc-tagged PTK7 deletion construct lacking the kinase homology domain (∆kPTK7-MT) as well as a full-length HA-tagged Ror2 construct were expressed in MCF7 cells. **B** Constructs and their protein domains are depicted in the top panel. Abbreviations are as follows: IG (immunoglobulin domain), CRD (cysteine-rich domain), K (kringle domain), TM (transmembrane domain), KH (kinase homology domain), TK (tyrosine kinase), S/T (serine/threonine-rich domain), P (proline-rich domain). **C** Immunoprecipitation experiment; the cell transfection scheme is indicated at the top. Co-immunoprecipitation was carried out using either anti-myc (IP **α**-MT, upper panel) or anti-HA antibodies (IP **α**-HA, middle panel). The respective cell lysates are shown in the bottom panel. Antibodies used for Western blotting and molecular weights are indicated at the right.

Published data suggest that PTK7 is a versatile co-receptor, however, its interaction partners in migrating NC cells have not been determined yet. To analyze if PTK7 may interact with the Ror2 receptor, we first checked for co-localization in migrating NC cells. RFP-labeled PTK7 and GFP-labeled Ror2 were expressed in *Xenopus* NC cells, which were explanted at premigratory stage 17 and monitored using live-cell imaging. Interestingly, distinct areas of the cell membrane could be distinguished where either PTK7, Ror2 or a combination of both proteins were localized (Fig 1A). The fact that PTK7 and Ror2 co-localize at the cell membrane suggests that these proteins may form a co-receptor complex.

In order to determine a potential biochemical interaction between PTK7 and Ror2 we tested for co-immunoprecipitation. Full-length myc-tagged PTK7 (PTK7-MT) and full-length HA-tagged Ror2 (Ror2-HA) were overexpressed in MCF7 cells. Precipitation of PTK7 using myc-antibodies resulted in co-precipitation of Ror2, while EGFR and TGFßR1 were not co-precipitated (Fig 1B and 1C; S1 Fig). Conversely, precipitation of Ror2 using HA-antibodies co-precipitated PTK7 (Fig 1C), but not EGFR or TGFßR1 (S1 Fig). As the intracellular kinase-homology domain of PTK7 is also required for its function [13,30], we further tested if full-length Ror2 also interacts with a PTK7 construct lacking this domain (∆kPTK7-HA). Indeed, we found that Ror2 co-precipitates ∆kPTK7 and vice versa (Fig 1C). Thus, these data suggest that PTK7 and Ror2 interact and that this interaction does not require the kinase homology domain of PTK7.

**Fig 2:**
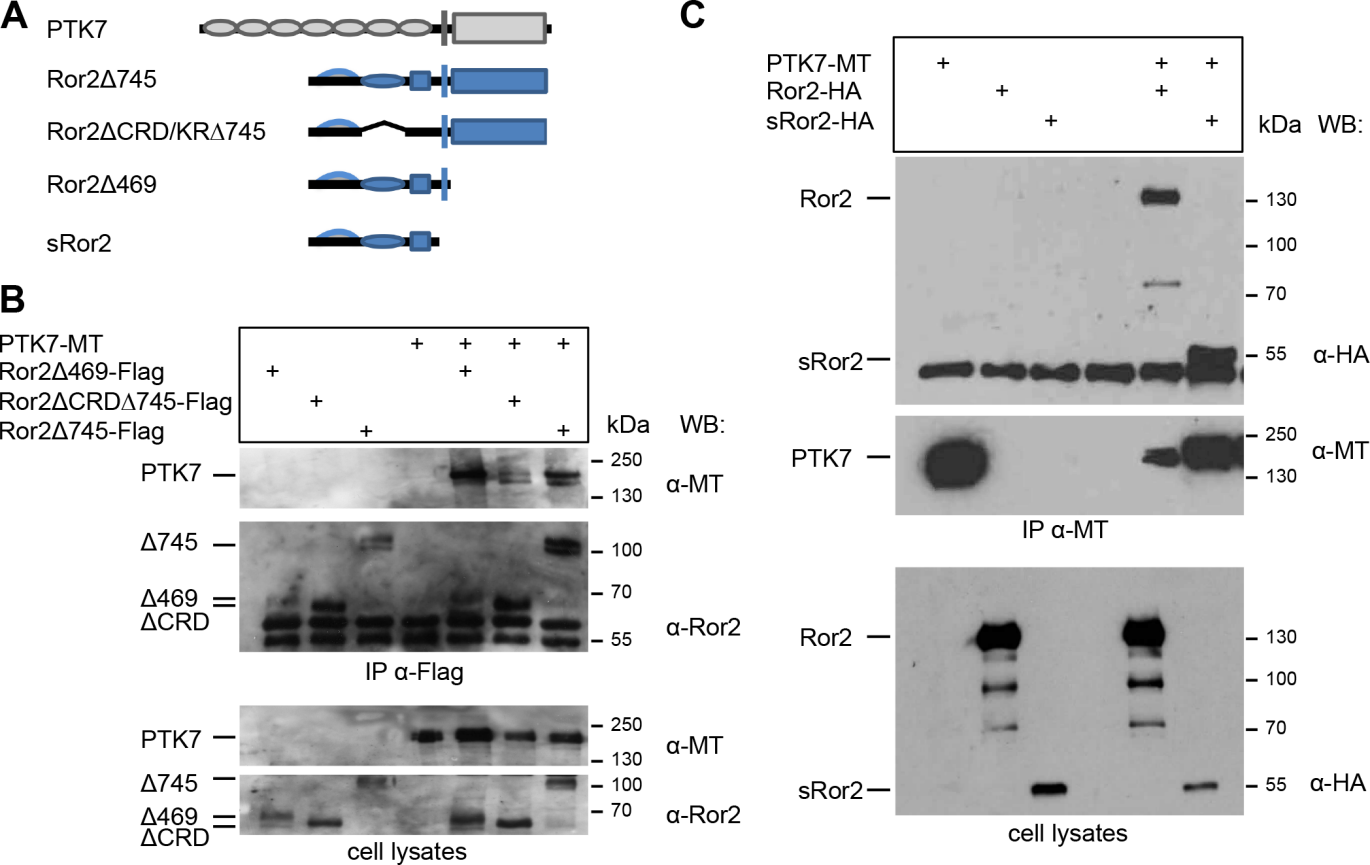
The intracellular domain and the CRD domain of Ror2 are not required for PTK7/Ror2 interaction. Myc-tagged PTK7 (PTK7-MT) together with FLAG- or HA-tagged Ror2 deletions were expressed in MCF7 cells. **A** The respective constructs and their protein domains are depicted in the top panel. **B** Immunoprecipitation using anti-FLAG antibodies; the cell transfection scheme is indicated at the top. Co-precipitated PTK7 was detected using anti-myc antibodies. Immunoprecipitated PTK7 is shown in the top panel, immunoprecipitated Ror2 constructs in the middle panel and cell lysates in the bottom panel. Antibodies used for Western blotting and molecular weights are indicated at the right. **C** Immunoprecipitation using anti-myc antibodies; the cell transfection scheme is indicated at the top. Co-precipitated full-length Ror2 and sRor2 were detected using anti-HA antibodies (top panel). Immunoprecipitated PTK7 is shown in the middle panel and cell lysates in the bottom panel. Antibodies used for Western blotting and molecular weights are indicated at the right.

To determine which Ror2 domains are required for PTK7/Ror2 interaction different Ror2 deletion mutants were used (Fig 2A). Ror2Δ745 has a deletion of the intracellular serine/threonine-rich domains. The Ror2ΔCRD/KR∆745 mutant lacks additionally the extracellular frizzled-like cysteine-rich domain (CRD) required for Wnt binding and the kringle domain (KR). In the Ror2Δ469 construct major parts of the intracellular domain including the tyrosine-kinase domain, the serine/threonine-rich and the proline-rich domains have been deleted. Finally, the sRor2 lacks the entire intracellular and transmembrane domains. The FLAG-tagged Ror2 deletion constructs were co-expressed with full-length myc-tagged PTK7 and co-precipitations were performed using anti-FLAG antibodies. All Ror2 deletion mutants were able to co-precipitate full-length PTK7 (Fig 2B and 2C and data not shown). Conversely, if PTK7 was precipitated using anti-myc antibodies sRor2 was co-precipitated (Fig 2C). Thus, the PTK7/Ror2 interaction is likely mediated by the extracellular domains of these proteins, but does not require the CRD and kringle domains of Ror2.

### PTK7 loss of function inhibits cell motility of explanted NC cells

**Fig 3:**
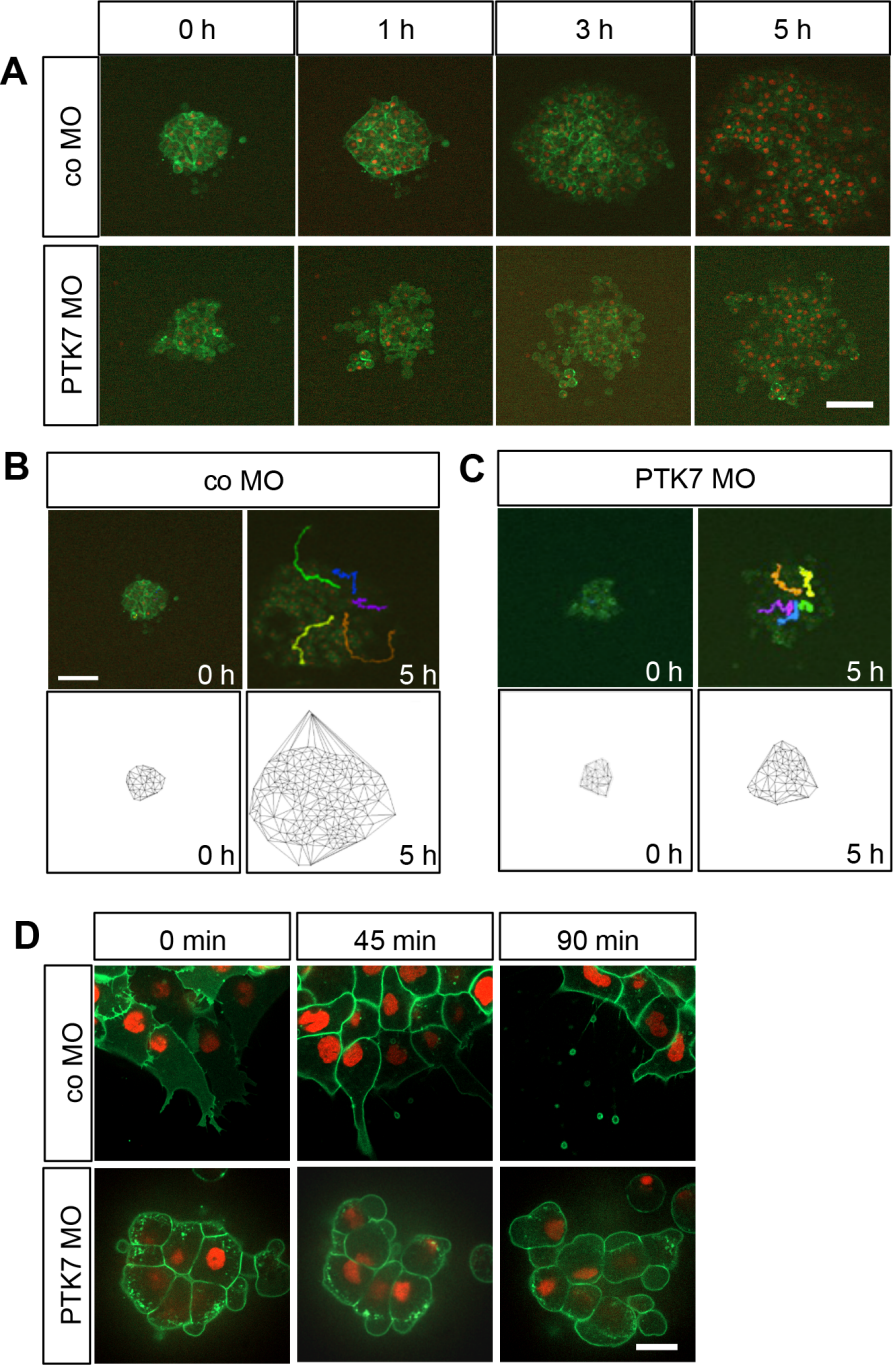
Loss of function of PTK7 affects NC cell shape and inhibits migration of explanted NC. **A** Time series showing explanted NC cells injected with 7.5 ng control or PTK7 MO in combination with 50 pg *mGFP* RNA and 250 pg *H2B-mcherry*. Cranial NC explants were excised at stage 16–17 and explanted on a fibronectin matrix and incubated until they had stably adhered to the matrix. NC migration was monitored for 5 hours using spinning disk microscopy (10x objective NA 0.45). Images for representative explants injected either with control or PTK7 MO are shown at the start of the experiment (0 h) or after 1, 3 or 5 hours; scale bar = 50 μm. **B** Cell tracking and Delaunay triangulation for explants injected with 7.5 ng co MO. The upper panel shows a single frame of the spinning disk movie and the lower panel the Delaunay triangulation at the start of the experiment (0 h) or after 5 hours. Cells were tracked over the whole five-hour time interval using the H2B staining of single nuclei. These tracks are shown for single cells as differently colored lines in the images taken after 5 hours, scale bar = 50 μm. **C** Cell tracking and Delaunay triangulation for explants injected with 7.5 ng PTK7 MO. **D** Time series showing explants injected with 7.5 ng co MO (upper panel) or 7.5 ng PTK7 MO (lower panel) at a higher magnification. Injected NC cells were explanted at stage 17, cultured for 1.5 hours and imaged with a 63x objective (NA 1.4); scale bar = 20 μm. Images are shown at the start of the experiment (0 min) and after 45 and 90 minutes.

As we detected co-localization of PTK7 and Ror2 in explanted NC cells, we used this system to further evaluate the biological relevance of the PTK7/Ror2 interaction. In *Xenopus* PTK7 has been shown to be required for NC migration [13], but its function on a cellular level remains elusive. Therefore, we used live-cell imaging to further analyze the migration behavior of explanted control and PTK7 morphant NC cells. Membrane-targeted GFP (mGFP) and cherry-labeled Histone2B (H2B-mcherry) were co-expressed to visualize cell shape and polarity. At the start of the experiment (0 hours), control NC cells formed a cell cluster where the leading-edge cells started to get polarized (Fig 3A, upper panel, S1 Movie). After one hour the leading cells left the main cell cluster and then dispersed rapidly during the 5 hour time-span of the experiment. In contrast, PTK7 MO injected NC cells adopted a more roundish shape and did not disperse as well as control cells (Fig 3 A, lower panel, S1 Movie). The dispersion of NC cells can be seen in the time series (Fig 3A), where–although explant size in the different experimental conditions is comparable at the beginning of the time-series–the area of cell spreading is significantly larger for the controls than the MO-injected explants after 5 hours of imaging. As it is difficult to determine the exact initial cell number and as the cell dissociation may not occur homogenously, we used cell tracking and Delaunay triangulation as more sophisticated methods to measure cell dispersion (Fig 3B and 3C). Delaunay triangulation determines the two closest cell neighbors and the area of the formed triangles is proportional to the cell dispersion. The time series as well as cell tracking and Delaunay triangulation all show that control cells dispersed efficiently during the 5 hours of imaging (Fig 3B). In contrast, in explants where PTK7 protein expression was knocked down, the NC cells had difficulties to detach from the main cluster and did not disperse as well as the control NC cells (Fig 3C). Higher magnification shows that PTK7 morphant cells do not form extensive protrusions like control cells, but adopt a more roundish shape. This shape change does not correlate with cell death as determined by Propidium iodine staining (S2 Fig). Some of these explants are still able to form protrusion, but others adopt a cell movement reminiscent of blebbing (Fig 3D, S2 Movie). Thus, PTK7 loss of function has severe effects on NC cell shape and motility.

### Ror2 rescues the PTK7 loss of function NC migration defect and this requires the kinase domain of Ror2

**Fig 4:**
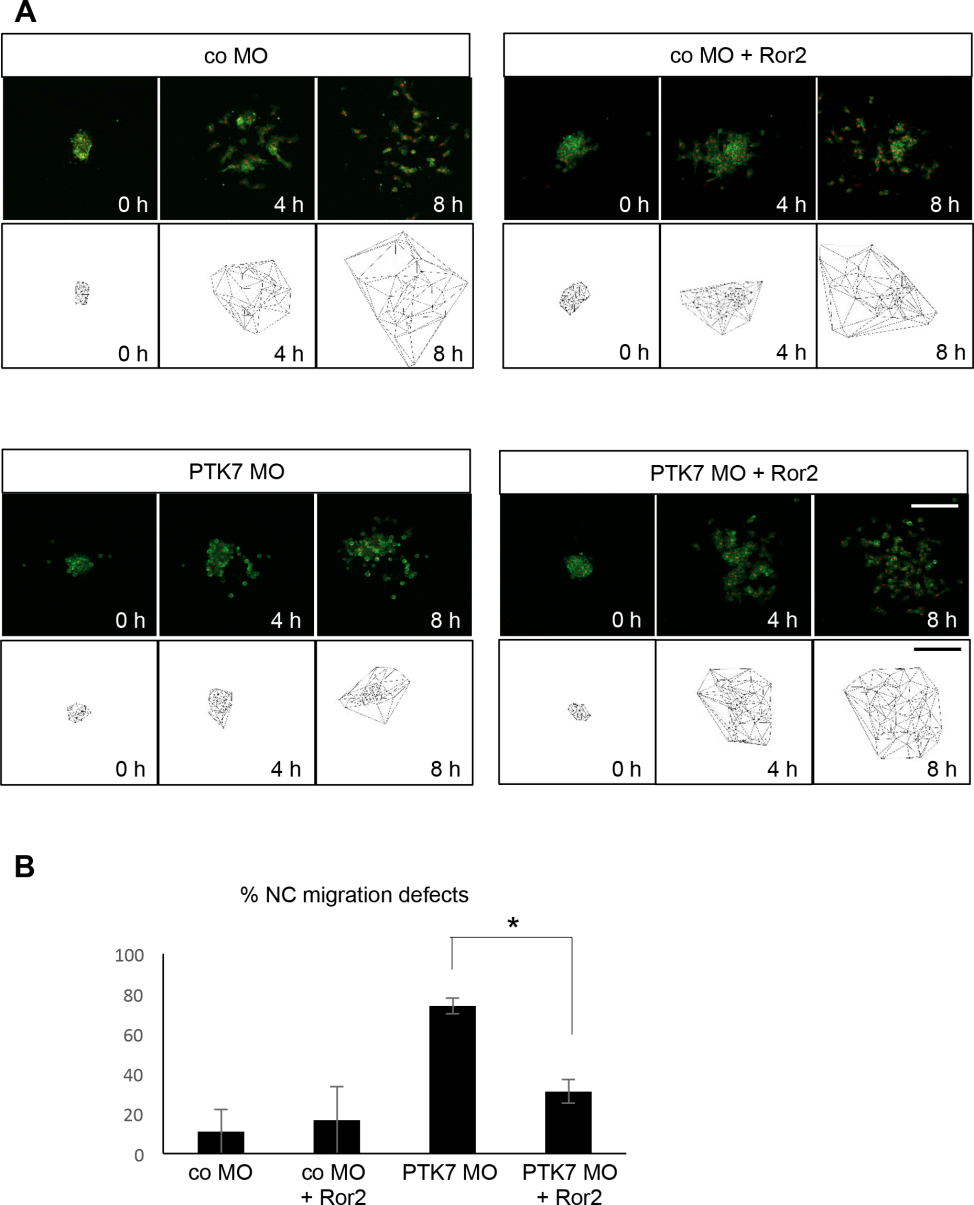
Ror2 rescues the PTK7 loss of function phenotype in explanted NC cells. **A** NC explants injected with 7.5 ng MO in combination with 50 pg *mGFP* RNA, 250 pg *H2B-mcherry* and 150 pg *Ror2* RNA. Time-lapse images (upper panel) and Delaunay triangulations (lower panel) at the start of the experiment (0 h) and after 4 or 8 hours are shown for the different conditions. **B** Graph summarizing percentage of migration defects of 3 independent experiments (total of 39 explants). Standard error of the means are shown. Asterisks indicates a p-value in a Student’s t-test < 0.05. Scale bars = 200 μm.

As PTK7 and Ror2 are both non-canonical Wnt receptors, which share functions in Wnt signaling, we analyzed if Ror2 can rescue the PTK7 loss of function NC migration defect. To this end NC cells injected either with co MO or PTK7 MO alone or in combination with Ror2 were explanted and their migration was analyzed by time-lapse imaging. While the majority of PTK7 MO injected NC cells showed migration defects, NC cells, which were co-injected with the PTK7 MO and Ror2 showed normal NC migration behavior (Fig 4, S3 Movie). Defects in protrusion formation as well as cell motility were rescued by co-expression of Ror2 (Fig 4A and 4B, S3 Movie) indicating that Ror2 expression can substitute for PTK7 in NC cells.

**Fig 5:**
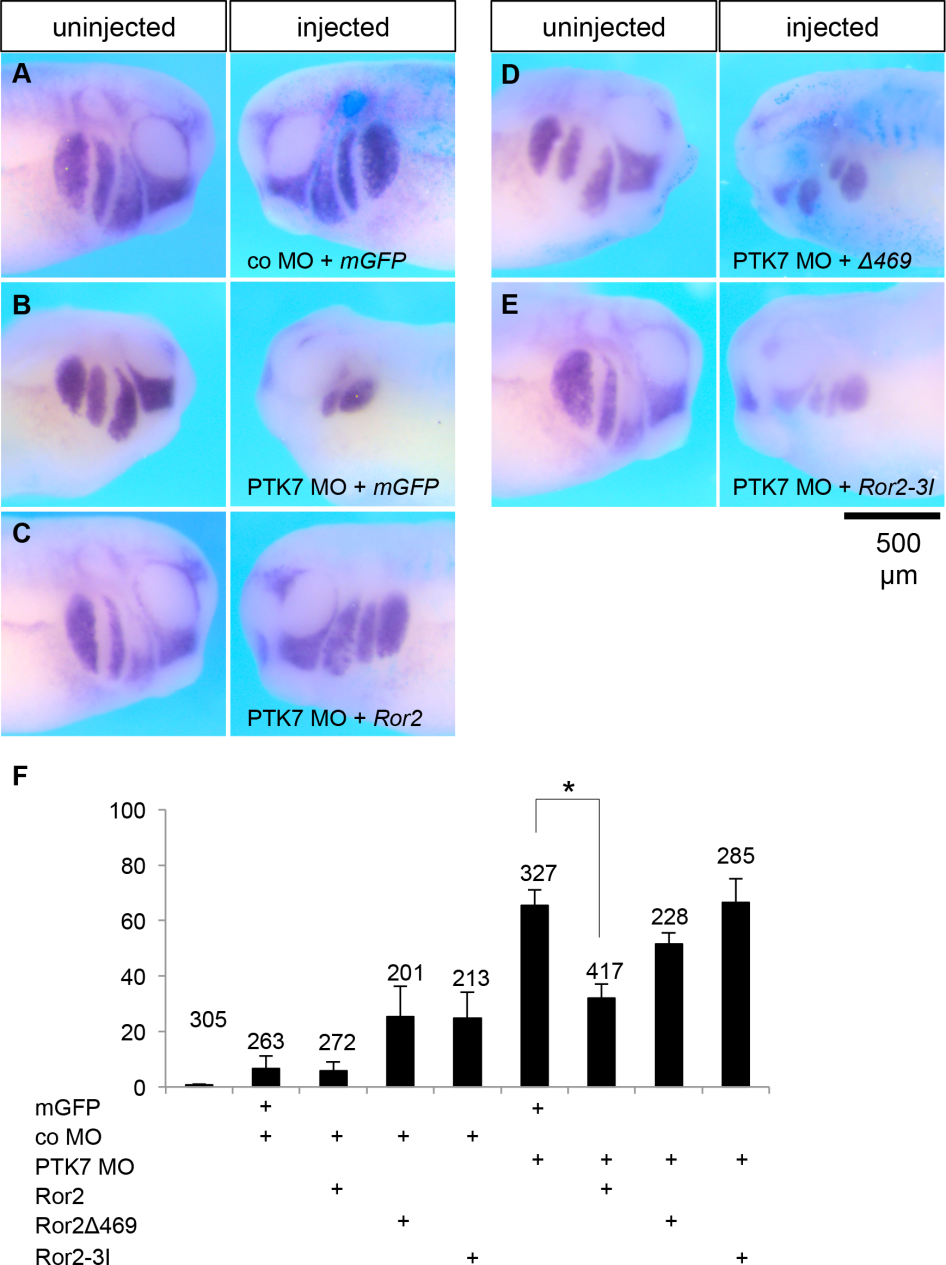
The kinase domain of Ror2 is required to rescue the NC migration defect in PTK7 morphant embryos. *Xenopus* embryos were injected with different constructs in combination with 100 pg *LacZ* RNA as a lineage tracer and analyzed by whole-mount *in situ* hybridization using a *twist* antisense RNA probe. **A** Embryo injected with 10 ng control MO and 100 pg *GFP* RNA shows normal NC migration. **B** Embryo injected with 10 ng PTK7 MO and 100 pg *GFP* RNA shows inhibition of NC migration on the injected side, while NC migration is normal on the uninjected side. **C** Co-injection of 10 ng PTK7 MO together with 100 pg *Ror2* RNA rescues the NC migration defect. **D** Embryo injected with 10 ng PTK7 MO and 100 pg of *Ror2Δ469* RNA. The embryo shows a NC migration defect on the injected side. **E** Embryo injected with PTK7 MO and a kinase dead mutant of Ror2 (Ror2-3I) showing a NC migration defect on the injected side. **F** Graph summarizing the percentage of NC migration defects of a minimum of 5 independent experiments for each experimental condition. Asterisks indicates a p-value in a Student’s t-test < 0.001. Scale bar = 500 μm.

Similar results were obtained in *in vivo* experiments where NC migration was analyzed at tadpole stages using twist *in situ* hybridization. Embryos injected with co MO were unaffected, while the majority of embryos injected with the PTK7 MO showed severe NC migration defects (Fig 5A, 5B and 5F). Co-injection of *Ror2* RNA with the PTK7 MO significantly decreased the percentage of embryos showing NC migration defects (Fig 3C and 3F). In contrast co-injection of a deletion mutant of *Ror2* lacking major parts of the intracellular domain, *Ror2Δ469* (Fig 2A and 2F) did not show a significant rescue defect (Fig 3D and 3F). Interestingly, a kinase dead mutant of Ror2 (Ror2-3I), where three lysines at position 504 (in the putative ATP-binding motif), 507 and 509 were all replaced with isoleucine [57] to abolish the catalytic activity, was not able to rescue the PTK7 morphant NC migration defect. Thus, these results suggest that Ror2 can functionally replace PTK7 and that the kinase function of Ror2 is required for this activity.

## Discussion

The PTK7 receptor functions in various processes ranging from embryonic morphogenesis to wound repair and its distinct functions are likely regulated by receptor context. PTK7 has been shown to interact with Frizzled7 and LRP6 ([13,35,38], however, an interaction with bona fide non-canonical Wnt receptors has so far not been analyzed. Concerning the PTK7 receptor complex our data suggest that PTK7 may interact with the non-canonical Ror2 Wnt-receptor. Like PTK7, Ror2 is expressed in migrating NC cells [57,60] and a role in NC development has been suggested [69]. Further, using fluorescently labeled PTK7 and Ror2 we detect a co-localization of both proteins in NC cells. Co-immunoprecipitation experiments show an interaction of Ror2 with PTK7 and suggest the extracellular domains as possible sites of interaction. More specifically, we observed that a Ror2 mutant lacking most of the extracellular part except the Ig-like domain was sufficient for binding to PTK7. Both, PTK7 and Ror2 possess Ig-like domains, which mediate a large variety of protein-protein interactions (for review see [70]). However, we can currently not rule out that there may be additional interaction sites. As co-immunoprecipitation experiments were performed in cell lysates it remains unclear if this interaction is direct or for example mediated by Wnt ligands or Frizzled co-receptors. Since interaction of both PTK7 and Ror2 with Frizzled receptors has been shown [13,36,50,55], members of the Frizzled receptor family may also contribute to the PTK7/Ror2 interaction. In addition, PTK7 [35,36] and Ror2 interact with different members of the Wnt protein family, although in the case of Ror2 only non-canonical Wnts such as Wnt-5a were found to activate downstream signaling [49,50,57,71]. Thus, although the exact molecular composition of a PTK7/Ror2 receptor complex remains yet to be analyzed, the formation and composition may also affect the selective binding to members of the Wnt family and thereby modulate intracellular signaling events.

The downstream signaling events of the PTK7/Ror2 complex are currently unclear, but may be mediated by an activation of c-Jun N-terminal kinase (JNK). Previously, we have shown that PTK7 overexpression in *Xenopus* ectodermal explants leads to a nuclear localization of phosphorylated JNK [13] and this is not observed if a kinase deletion mutant of PTK7 (∆kPTK7) is overexpressed (data not shown). Ror2 has been shown to have a similar function [44,46,53]. Therefore, possibly these molecules collaborate to recruit Dishevelled and to activate JNK via the PCP pathway [72] and thereby enable the migration of NC cells [8,9,17,18,25]. Supporting this hypothesis, Ror2 overexpression can rescue the PTK7 loss of function NC migration defect possibly by compensating for PTK7. Consistently, this rescue effect requires the kinase domain of Ror2, which is necessary for JNK activation [44]. In the context of mesodermal convergent extension it was previously shown that Ror2 activates a signaling cascade including PI3K, the small GTPase cdc42, MKK7, JNK and c-jun and ATF2 transcription factors [44]. For PTK7 the mechanism of JNK activation remains unclear, but may involve the adaptor protein RACK1, which is required for PTK7-mediated Dishevelled recruitment [30]. As RACK1 plays a role in PKC-mediated JNK phosphorylation [73], PTK7 may activate JNK via RACK1 or Dishevelled itself [72]. JNK is involved in a multitude of processes and can activate the transcription factors jun, fos and ATF2 [74]. Like Ror2, PTK7 is also able to activate ATF2-mediated transcription as shown by activation of an ATF2 luciferase reporter in *Xenopus* lysates [35,75]. Thus, one of the common signaling outcomes of PTK7 and Ror2 may be the transcription of ATF2-dependent target genes, which has previously also been acknowledged as a readout for a bona fide activator of non-canonical Wnt signaling [75].

In addition to activation of JNK, PTK7 and Ror2 share a function in the inhibition of canonical Wnt signaling [34,35,45,53], which may contribute to their role in NC development. The molecular mechanisms are currently unclear. Ror2 may inhibit canonical Wnt signaling by regulating the stability of ß-catenin [49] or Tcf/Lef-dependent transcription [45,53]. Furthermore, it has been suggested that formation of a non-canonical Wnt5a/Ror2/Frizzled complex could compete for available Frizzled receptors thereby inhibiting the formation of a canonical Wnt3a/LRP/Frizzled complex [47] and subsequently canonical Wnt signaling. PTK7 likely inhibits canonical Wnt signaling by a similar mechanism. As PTK7 can form a complex with canonical Wnt ligands and Frizzled7 [35], it could also sequester Frizzled7 in a non-canonical Wnt signaling complex thereby preventing its interaction with canonical co-receptors and Wnt ligands. Indeed, it has been shown that Frizzled7 functions in canonical and non-canonical Wnt signaling depending on Wnt- and likely also co-receptor-context [76]. Independent of the mechanism by which PTK7 or Ror2 inhibit canonical Wnt signaling, this activity could explain their function in NC migration. Previously, it has been shown that ectopic activation of canonical Wnt signaling inhibits NC migration. Explanted NC cells treated with LiCl, an inhibitor of GSK3ß, or exogenous Wnt1 show an inhibition of NC migration [77]. Thus, loss of function of PTK7 could enhance canonical Wnt signaling thereby inhibiting NC migration. As Ror2 can also inhibit canonical Wnt signaling, this may account for its ability to substitute for PTK7 in NC cells. Currently it remains unclear by which mechanism a PTK7/Ror2 complex affects NC migration. We have shown that the tyrosine kinase domain of Ror2 is required to rescue PTK7 loss of function. However, as the tyrosine kinase activity of Ror2 is required for both activation of JNK and inhibition of canonical Wnt signaling [44,78], Ror2 is capable to compensate for both putative functions of PTK7. Therefore, it remains to be seen which one of these or if possibly even a combination of both is required. In summary, PTK7 and Ror2 share signaling functions that may be enhanced by the combination of both molecules and that may allow for example Ror2 to compensate for PTK7.

A possible interaction of PTK7 with Ror2 is likely not limited to NC cells, as PTK7 and Ror2 have also been implicated in tumor development and progression. PTK7 and Ror2 expression is frequently deregulated in a variety of cancers [79,80,81,82,83,84,85]. Several studies indicate an association of Ror2, in some cases downstream of Wnt5a, with tumor invasiveness and metastasis [82,84,86,87,88]. However, it has been shown that epigenetic silencing of Ror2 in colon cancer promotes cell proliferation and tumor growth [89], suggesting that depending on the cellular context Ror2 could also act as a tumor-suppressor. Similarly, contradictory results are also seen for the function of PTK7. PTK7 is expressed in acute myeloid leukemia, where it promotes cell migration and leads to a poor clinical outcome [90]. Additional studies found migration and invasion promoting functions of PTK7 in lung cancer, intrahepatic cholangiocarcinoma, glioma and prostate cancer [42,91,92,93]. In contrast, another publication shows that PTK7 is a target of membrane type-1 matrix metalloproteinase and that PTK7 expression inhibits cell invasion [94]. Thus, the function of PTK7 and Ror2 may depend on tumor context and ultimately on receptor context.

## Supporting Information

S1 Fig PTK7 and Ror2 co-precipitate each other but not EGFR or TGFß1R. **A** Full-length myc-tagged PTK7 (PTK7-MT) was co-expressed with Ror2-EGFP, EGFR-EGFP [95] or TGFß1R-EGFP (kind gift of A. Menke, Molecular Oncology of Solid Tumors, Giessen, Germany) as indicated in MCF7 cells. Cell lysates were precipitated using anti-myc antibodies (IP **α**-MT, upper panel). Precipitates are shown in the upper panels, cell lysates in the lower panel. Antibodies used for Western blotting and molecular weights are indicated at the right. **B** Full-length HA-tagged Ror2 was co-expressed with PTK7-MT, EGFR-EGFP or TGFß1R-GFP as indicated in MCF7 cells and cell lysates were precipitated using anti-HA antibodies (IP **α**-HA, upper panel). Precipitates are shown in the three upper panels and lysates in the two lower panels. Antibodies used for Western blotting and molecular weights are indicated at the right; * marks unspecific bands, ** Ror2 signal remaining from previous anti-HA staining, which was only partially removed by blot stripping.
(TIF)

S2 Fig Propidium Iodine (PI) staining to determine the viability of PTK7 loss of function NC cells. NC explants injected with 7.5 ng control MO or PTK7 MO in combination with 50 pg mGFP were treated with PI (10 μg/ml) to test for the viability of the explanted NC cells. Cell membrane integrity prevents PI staining of viable cells, while it can stain nucleic acids of apoptotic cells (red). In PTK7 morphant cells few PI-positive cells appear after 3 hours compared to 6 hours in controls (one example each is marked by a red arrow). Round-shaped PTK7 morphant NC cells appear early in the experiment and keep moving/blebbing for up to a couple of hours (white arrows, numbers indicate specific cells during the course of the experiment). Thus, the roundish cell shape is not necessarily an indication of cell death. Dashed squares show higher magnifications of specific cells. Scale bar = 50 μm.
(TIF)

S1 Movie Time-lapse movie showing the migration of NC cells injected with control MO (left) in comparison to an explant injected with PTK7 MO. Images were taken using a Spinning Disk microscope with a 10x plan apochromat objective NA (0.45).
(AVI)

S2 Movie Higher magnification using a 63x plan apochromat objective NA 1.4) of control MO (left) and PTK7 MO injected (right) NC explants. (AVI)

S3 Movie Time-lapse movie showing that Ror2 expression rescues the PTK7 morphant phenotype. Explants injected with control MO (upper panel, left), control MO and Ror2 RNA (upper panel, right), PTK7 MO (lower panel, left) or PTK7 MO and Ror2 RNA (loer panel, right) are shown over a time interval of 8 hours and 20 minutes.
(AVI)

## References

[1] Powell DR, Blasky AJ, Britt SG, Artinger KB (2013) Riding the crest of the wave: parallels between the neural crest and cancer in epithelial-to-mesenchymal transition and migration. Wiley interdisciplinary reviews Systems biology and medicine 5: 511–522. 10.1002/wsbm.1224 23576382 PMC3739939

[2] Kuriyama S, Mayor R (2008) Molecular analysis of neural crest migration. Philos Trans R Soc Lond B Biol Sci. 10.1098/rstb.2007.2252 PMC2610123 18198151

[3] Theveneau E, Mayor R (2012) Neural crest delamination and migration: from epithelium-to-mesenchyme transition to collective cell migration. Developmental biology 366: 34–54. 10.1016/j.ydbio.2011.12.041 22261150

[4] Theveneau E, Mayor R (2011) Collective cell migration of the cephalic neural crest: the art of integrating information. Genesis 49: 164–176. 10.1002/dvg.20700 21157935

[5] Snider TN, Mishina Y (2014) Cranial neural crest cell contribution to craniofacial formation, pathology, and future directions in tissue engineering. Birth defects research Part C, Embryo today: reviews 102: 324–332. 10.1002/bdrc.21075 PMC4320944 25227212

[6] Zhang D, Ighaniyan S, Stathopoulos L, Rollo B, Landman K, Hutson J, et al (2014) The neural crest: a versatile organ system. Birth defects research Part C, Embryo today: reviews 102: 275–298. 10.1002/bdrc.21081 25227568

[7] Bolande RP (1997) Neurocristopathy: its growth and development in 20 years. Pediatric pathology & laboratory medicine: journal of the Society for Pediatric Pathology, affiliated with the International Paediatric Pathology Association 17: 1–25. 9050057

[8] Carmona-Fontaine C, Matthews HK, Kuriyama S, Moreno M, Dunn GA, Parsons M, et al (2008) Contact inhibition of locomotion in vivo controls neural crest directional migration. Nature 456: 957–961. 10.1038/nature07441 19078960 PMC2635562

[9] Matthews HK, Marchant L, Carmona-Fontaine C, Kuriyama S, Larrain J, Holt MR, et al (2008) Directional migration of neural crest cells in vivo is regulated by Syndecan-4/Rac1 and non-canonical Wnt signaling/RhoA. Development 135: 1771–1780. 10.1242/dev.017350 18403410

[10] Theveneau E, Steventon B, Scarpa E, Garcia S, Trepat X, Streit A, et al (2013) Chase-and-run between adjacent cell populations promotes directional collective migration. Nature cell biology 15: 763–772. 10.1038/ncb2772 23770678 PMC4910871

[11] Banerjee S, Gordon L, Donn TM, Berti C, Moens CB, Burden SJ, et al (2011) A novel role for MuSK and non-canonical Wnt signaling during segmental neural crest cell migration. Development 138: 3287–3296. 10.1242/dev.067306 21750038 PMC3133918

[12] Ulmer B, Hagenlocher C, Schmalholz S, Kurz S, Schweickert A, Kohl A, et al (2013) Calponin 2 acts as an effector of noncanonical Wnt-mediated cell polarization during neural crest cell migration. Cell reports 3: 615–621. 10.1016/j.celrep.2013.02.015 23499442

[13] Shnitsar I, Borchers A (2008) PTK7 recruits dsh to regulate neural crest migration. Development 135: 4015–4024. 10.1242/dev.023556 19004858

[14] Mayor R, Theveneau E (2014) The role of the non-canonical Wnt-planar cell polarity pathway in neural crest migration. The Biochemical journal 457: 19–26. 10.1042/BJ20131182 24325550

[15] Abercrombie M, Heaysman JE (1953) Observations on the social behaviour of cells in tissue culture. I. Speed of movement of chick heart fibroblasts in relation to their mutual contacts. Experimental cell research 5: 111–131. 13083622 10.1016/0014-4827(53)90098-6

[16] Abercrombie M, Heaysman JE (1954) Observations on the social behaviour of cells in tissue culture. II. Monolayering of fibroblasts. Exp Cell Res 6: 293–306. 13173482 10.1016/0014-4827(54)90176-7

[17] Theveneau E, Marchant L, Kuriyama S, Gull M, Moepps B, Parsons M, et al (2010) Collective chemotaxis requires contact-dependent cell polarity. Developmental cell 19: 39–53. 10.1016/j.devcel.2010.06.012 20643349 PMC2913244

[18] Mayor R, Carmona-Fontaine C (2010) Keeping in touch with contact inhibition of locomotion. Trends in cell biology 20: 319–328. 10.1016/j.tcb.2010.03.005 20399659 PMC2927909

[19] Axelrod JD, McNeill H (2002) Coupling planar cell polarity signaling to morphogenesis. ScientificWorldJournal 2: 434–454. 12806028 10.1100/tsw.2002.105 PMC6009572

[20] Klein TJ, Mlodzik M (2005) Planar cell polarization: an emerging model points in the right direction. Annu Rev Cell Dev Biol 21: 155–176. 16212491 10.1146/annurev.cellbio.21.012704.132806

[21] Vladar EK, Antic D, Axelrod JD (2009) Planar cell polarity signaling: the developing cell's compass. Cold Spring Harbor perspectives in biology 1: a002964 10.1101/cshperspect.a002964 20066108 PMC2773631

[22] Wallingford JB (2012) Planar cell polarity and the developmental control of cell behavior in vertebrate embryos. Annual review of cell and developmental biology 28: 627–653. 10.1146/annurev-cellbio-092910-154208 22905955

[23] Theveneau E, Mayor R (2010) Integrating chemotaxis and contact-inhibition during collective cell migration: Small GTPases at work. Small GTPases 1: 113–117. 21686264 10.4161/sgtp.1.2.13673 PMC3116595

[24] Clay MR, Halloran MC (2013) Rho activation is apically restricted by Arhgap1 in neural crest cells and drives epithelial-to-mesenchymal transition. Development 140: 3198–3209. 10.1242/dev.095448 23804498 PMC3931734

[25] Theveneau E, Mayor R (2013) Collective cell migration of epithelial and mesenchymal cells. Cellular and molecular life sciences: CMLS 70: 3481–3492. 10.1007/s00018-012-1251-7 23314710 PMC11113167

[26] Becker SF, Mayor R, Kashef J (2013) Cadherin-11 mediates contact inhibition of locomotion during Xenopus neural crest cell migration. PloS one 8: e85717 10.1371/journal.pone.0085717 24392028 PMC3877381

[27] Carmona-Fontaine C, Theveneau E, Tzekou A, Tada M, Woods M, Page KM, et al (2011) Complement fragment C3a controls mutual cell attraction during collective cell migration. Developmental cell 21: 1026–1037. 10.1016/j.devcel.2011.10.012 22118769 PMC3272547

[28] Miller MA, Steele RE (2000) Lemon encodes an unusual receptor protein-tyrosine kinase expressed during gametogenesis in Hydra. Dev Biol 224: 286–298. 10926767 10.1006/dbio.2000.9786

[29] Kroiher M, Miller MA, Steele RE (2001) Deceiving appearances: signaling by "dead" and "fractured" receptor protein-tyrosine kinases. Bioessays 23: 69–76. 11135311 10.1002/1521-1878(200101)23:1<69::AID-BIES1009>3.0.CO;2-K

[30] Wehner P, Shnitsar I, Urlaub H, Borchers A (2011) RACK1 is a novel interaction partner of PTK7 that is required for neural tube closure. Development 138: 1321–1327. 10.1242/dev.056291 21350015

[31] Lu X, Borchers AG, Jolicoeur C, Rayburn H, Baker JC, Tessier-Lavigne M (2004) PTK7/CCK-4 is a novel regulator of planar cell polarity in vertebrates. Nature 430: 93–98. 15229603 10.1038/nature02677

[32] Yen WW, Williams M, Periasamy A, Conaway M, Burdsal C, Keller R, et al (2009) PTK7 is essential for polarized cell motility and convergent extension during mouse gastrulation. Development 136: 2039–2048. 10.1242/dev.030601 19439496 PMC2685725

[33] Paudyal A, Damrau C, Patterson VL, Ermakov A, Formstone C, Lalanne Z, et al (2010) The novel mouse mutant, chuzhoi, has disruption of Ptk7 protein and exhibits defects in neural tube, heart and lung development and abnormal planar cell polarity in the ear. Bmc Developmental Biology 286: 20970–20976. 10.1186/1471-213X-10-87 PMC2930600 20704721

[34] Hayes M, Naito M, Daulat A, Angers S, Ciruna B (2013) Ptk7 promotes non-canonical Wnt/PCP-mediated morphogenesis and inhibits Wnt/beta-catenin-dependent cell fate decisions during vertebrate development. Development 140: 1807–1818. 10.1242/dev.090183 23533179

[35] Peradziryi H, Kaplan NA, Podleschny M, Liu X, Wehner P, Borchers A, et al (2011) PTK7/Otk interacts with Wnts and inhibits canonical Wnt signalling. The EMBO journal 30: 3729–3740. 10.1038/emboj.2011.236 21772251 PMC3173783

[36] Linnemannstons K, Ripp C, Honemann-Capito M, Brechtel-Curth K, Hedderich M, Wodarz A (2014) The PTK7-related transmembrane proteins off-track and off-track 2 are co-receptors for Drosophila Wnt2 required for male fertility. PLoS genetics 10: e1004443 10.1371/journal.pgen.1004443 25010066 PMC4091708

[37] Puppo F, Thome V, Lhoumeau AC, Cibois M, Gangar A, Lembo F, et al (2011) Protein tyrosine kinase 7 has a conserved role in Wnt/beta-catenin canonical signalling. EMBO reports 12: 43–49. 10.1038/embor.2010.185 21132015 PMC3024124

[38] Bin-Nun N, Lichtig H, Malyarova A, Levy M, Elias S, Frank D (2014) PTK7 modulates Wnt signaling activity via LRP6. Development 141: 410–421. 10.1242/dev.095984 24353057

[39] Andreeva A, Lee J, Lohia M, Wu X, Macara IG, Lu X (2014) PTK7-Src signaling at epithelial cell contacts mediates spatial organization of actomyosin and planar cell polarity. Developmental cell 29: 20–33. 10.1016/j.devcel.2014.02.008 24703874 PMC4086913

[40] Golubkov VS, Strongin AY (2014) Downstream signaling and genome-wide regulatory effects of PTK7 pseudokinase and its proteolytic fragments in cancer cells. Cell communication and signaling: CCS 12: 15 10.1186/1478-811X-12-15 24618420 PMC4007575

[41] Chen R, Khatri P, Mazur PK, Polin M, Zheng Y, Vaka D, et al (2014) A meta-analysis of lung cancer gene expression identifies PTK7 as a survival gene in lung adenocarcinoma. Cancer research 74: 2892–2902. 10.1158/0008-5472.CAN-13-2775 24654231 PMC4084668

[42] Jin J, Ryu HS, Lee KB, Jang JJ (2014) High expression of protein tyrosine kinase 7 significantly associates with invasiveness and poor prognosis in intrahepatic cholangiocarcinoma. PloS one 9: e90247 10.1371/journal.pone.0090247 24587299 PMC3938661

[43] Peradziryi H, Tolwinski NS, Borchers A (2012) The many roles of PTK7: a versatile regulator of cell-cell communication. Archives of biochemistry and biophysics 524: 71–76. 10.1016/j.abb.2011.12.019 22230326

[44] Schambony A, Wedlich D (2007) Wnt-5A/Ror2 regulate expression of XPAPC through an alternative noncanonical signaling pathway. Dev Cell 12: 779–792. 17488628 10.1016/j.devcel.2007.02.016

[45] Mikels AJ, Nusse R (2006) Purified Wnt5a protein activates or inhibits beta-catenin-TCF signaling depending on receptor context. Plos Biology 4: 570–582. 10.1371/journal.pbio.0040115 PMC1420652 16602827

[46] Nomachi A, Nishita M, Inaba D, Enomoto M, Hamasaki M, Minami Y (2008) Receptor tyrosine kinase Ror2 mediates Wnt5a-induced polarized cell migration by activating c-Jun N-terminal kinase via actin-binding protein filamin A. J Biol Chem 283: 27973–27981. 10.1074/jbc.M802325200 18667433

[47] Grumolato L, Liu GZ, Mong P, Mudbhary R, Biswas R, Arroyave R, et al (2010) Canonical and noncanonical Wnts use a common mechanism to activate completely unrelated coreceptors. Genes & Development 24: 2517–2530. 10.1101/gad.1957710 PMC2975928 21078818

[48] Winkel A, Stricker S, Tylzanowski P, Seiffart V, Mundlos S, Gross G, et al (2008) Wnt-ligand-dependent interaction of TAK1 (TGF-beta-activated kinase-1) with the receptor tyrosine kinase Ror2 modulates canonical Wnt-signalling. Cell Signal 20: 2134–2144. 10.1016/j.cellsig.2008.08.009 18762249

[49] Billiard J, Way DS, Seestaller-Wehr LM, Moran RA, Mangine A, Bodine PV (2005) The orphan receptor tyrosine kinase Ror2 modulates canonical Wnt signaling in osteoblastic cells. Molecular endocrinology 19: 90–101. 15388793 10.1210/me.2004-0153

[50] Oishi I, Suzuki H, Onishi N, Takada R, Kani S, Ohkawara B, et al (2003) The receptor tyrosine kinase Ror2 is involved in non-canonical Wnt5a/JNK signalling pathway. Genes to cells: devoted to molecular & cellular mechanisms 8: 645–654. 12839624 10.1046/j.1365-2443.2003.00662.x

[51] Kani S, Oishi I, Yamamoto H, Yoda A, Suzuki H, Nomachi A, et al (2004) The receptor tyrosine kinase Ror2 associates with and is activated by casein kinase Iepsilon. J Biol Chem 279: 50102–50109. 15375164 10.1074/jbc.M409039200

[52] Nishita M, Yoo SK, Nomachi A, Kani S, Sougawa N, Ohta Y, et al (2006) Filopodia formation mediated by receptor tyrosine kinase Ror2 is required for Wnt5a-induced cell migration. J Cell Biol 175: 555–562. 17101698 10.1083/jcb.200607127 PMC2064592

[53] Witte F, Bernatik O, Kirchner K, Masek J, Mahl A, Krejci P, et al (2010) Negative regulation of Wnt signaling mediated by CK1-phosphorylated Dishevelled via Ror2. Faseb J 24: 2417–2426. 10.1096/fj.09-150615 20215527

[54] Yamamoto H, Yoo SK, Nishita M, Kikuchi A, Minami Y (2007) Wnt5a modulates glycogen synthase kinase 3 to induce phosphorylation of receptor tyrosine kinase Ror2. Genes to cells: devoted to molecular & cellular mechanisms 12: 1215–1223. 17986005 10.1111/j.1365-2443.2007.01128.x

[55] Yamamoto S, Nishimura O, Misaki K, Nishita M, Minami Y, Yonemura S, et al (2008) Cthrc1 selectively activates the planar cell polarity pathway of Wnt signaling by stabilizing the Wnt-receptor complex. Dev Cell 15: 23–36. 10.1016/j.devcel.2008.05.007 18606138

[56] Gao B, Song H, Bishop K, Elliot G, Garrett L, English MA, et al (2011) Wnt signaling gradients establish planar cell polarity by inducing Vangl2 phosphorylation through Ror2. Developmental cell 20: 163–176. 10.1016/j.devcel.2011.01.001 21316585 PMC3062198

[57] Hikasa H, Shibata M, Hiratani I, Taira M (2002) The Xenopus receptor tyrosine kinase Xror2 modulates morphogenetic movements of the axial mesoderm and neuroectoderm via Wnt signaling. Development 129: 5227–5239. 12399314 10.1242/dev.129.22.5227

[58] He F, Xiong W, Yu X, Espinoza-Lewis R, Liu C, Gu S, et al (2008) Wnt5a regulates directional cell migration and cell proliferation via Ror2-mediated noncanonical pathway in mammalian palate development. Development 135: 3871–3879. 10.1242/dev.025767 18948417 PMC3010758

[59] Yamada M, Udagawa J, Matsumoto A, Hashimoto R, Hatta T, Nishita M, et al (2010) Ror2 is required for midgut elongation during mouse development. Developmental dynamics: an official publication of the American Association of Anatomists 239: 941–953. 20063415 10.1002/dvdy.22212

[60] Feike AC, Rachor K, Gentzel M, Schambony A (2010) Wnt5a/Ror2-induced upregulation of xPAPC requires xShcA. Biochem Biophys Res Commun. 10.1016/j.bbrc.2010.08.074 20732301

[61] Nieuwkoop PD, Faber J, Hubrecht Laboratory Utrecht. (1956) Normal table of Xenopus laevis (Daudin): a systematical and chronological survey of the development from the fertilized egg till the end of metamorphosis Amsterdam: North-Holland Pub. Co. 243 p. p.

[62] Smith WC, Harland RM (1991) Injected Xwnt-8 RNA acts early in Xenopus embryos to promote formation of a vegetal dorsalizing center. Cell 67: 753–765. 1657405 10.1016/0092-8674(91)90070-f

[63] Kim SH, Yamamoto A, Bouwmeester T, Agius E, Robertis EM (1998) The role of paraxial protocadherin in selective adhesion and cell movements of the mesoderm during Xenopus gastrulation. Development 125: 4681–4690. 9806917 10.1242/dev.125.23.4681

[64] Kashef J, Kohler A, Kuriyama S, Alfandari D, Mayor R, Wedlich D (2009) Cadherin-11 regulates protrusive activity in Xenopus cranial neural crest cells upstream of Trio and the small GTPases. Genes Dev 23: 1393–1398. 10.1101/gad.519409 19528317 PMC2701577

[65] Sammar M, Stricker S, Schwabe GC, Sieber C, Hartung A, Hanke M, et al (2004) Modulation of GDF5/BRI-b signalling through interaction with the tyrosine kinase receptor Ror2. Genes to cells: devoted to molecular & cellular mechanisms 9: 1227–1238. 15569154 10.1111/j.1365-2443.2004.00799.x

[66] Borchers A, David R, Wedlich D (2001) Xenopus cadherin-11 restrains cranial neural crest migration and influences neural crest specification. Development 128: 3049–3060. 11688555 10.1242/dev.128.16.3049

[67] Borchers A, Epperlein HH, Wedlich D (2000) An assay system to study migratory behavior of cranial neural crest cells in Xenopus. Dev Genes Evol 210: 217–222. 11180825 10.1007/s004270050307

[68] Meijering E, Dzyubachyk O, Smal I (2012) Methods for cell and particle tracking. Methods in enzymology 504: 183–200. 10.1016/B978-0-12-391857-4.00009-4 22264535

[69] Ossipova O, Sokol SY (2011) Neural crest specification by noncanonical Wnt signaling and PAR-1. Development 138: 5441–5450. 10.1242/dev.067280 22110058 PMC3222216

[70] Barclay AN (2003) Membrane proteins with immunoglobulin-like domains—a master superfamily of interaction molecules. Seminars in immunology 15: 215–223. 14690046 10.1016/s1044-5323(03)00047-2

[71] Liu Y, Rubin B, Bodine PV, Billiard J (2008) Wnt5a induces homodimerization and activation of Ror2 receptor tyrosine kinase. Journal of cellular biochemistry 105: 497–502. 10.1002/jcb.21848 18615587

[72] Boutros M, Paricio N, Strutt DI, Mlodzik M (1998) Dishevelled activates JNK and discriminates between JNK pathways in planar polarity and wingless signaling. Cell 94: 109–118. 9674432 10.1016/s0092-8674(00)81226-x

[73] Lopez-Bergami P, Habelhah H, Bhoumik A, Zhang W, Wang LH, Ronai Z (2005) RACK1 mediates activation of JNK by protein kinase C [corrected]. Mol Cell 19: 309–320. 16061178 10.1016/j.molcel.2005.06.025 PMC2953422

[74] Weston CR, Davis RJ (2002) The JNK signal transduction pathway. Current opinion in genetics & development 12: 14–21. 11790549 10.1016/s0959-437x(01)00258-1

[75] Ohkawara B, Niehrs C (2011) An ATF2-based luciferase reporter to monitor non-canonical Wnt signaling in Xenopus embryos. Dev Dyn 240: 188–194. 10.1002/dvdy.22500 21128306

[76] Medina A, Reintsch W, Steinbeisser H (2000) Xenopus frizzled 7 can act in canonical and non-canonical Wnt signaling pathways: implications on early patterning and morphogenesis. Mech Dev 92: 227–237. 10727861 10.1016/s0925-4773(00)00240-9

[77] de Melker AA, Desban N, Duband JL (2004) Cellular localization and signaling activity of beta-catenin in migrating neural crest cells. Developmental dynamics: an official publication of the American Association of Anatomists 230: 708–726. 15254905 10.1002/dvdy.20091

[78] Mikels A, Minami Y, Nusse R (2009) Ror2 receptor requires tyrosine kinase activity to mediate Wnt5A signaling. The Journal of biological chemistry 284: 30167–30176. 10.1074/jbc.M109.041715 19720827 PMC2781572

[79] Easty DJ, Mitchell PJ, Patel K, Florenes VA, Spritz RA, Bennett DC (1997) Loss of expression of receptor tyrosine kinase family genes PTK7 and SEK in metastatic melanoma. Int J Cancer 71: 1061–1065. 9185712 10.1002/(sici)1097-0215(19970611)71:6<1061::aid-ijc24>3.0.co;2-f

[80] Endoh H, Tomida S, Yatabe Y, Konishi H, Osada H, Tajima K, et al (2004) Prognostic model of pulmonary adenocarcinoma by expression profiling of eight genes as determined by quantitative real-time reverse transcriptase polymerase chain reaction. J Clin Oncol 22: 811–819. 14990636 10.1200/JCO.2004.04.109

[81] Muller-Tidow C, Schwable J, Steffen B, Tidow N, Brandt B, Becker K, et al (2004) High-throughput analysis of genome-wide receptor tyrosine kinase expression in human cancers identifies potential novel drug targets. Clin Cancer Res 10: 1241–1249. 14977821 10.1158/1078-0432.ccr-0954-03

[82] O'Connell MP, Fiori JL, Xu M, Carter AD, Frank BP, Camilli TC, et al (2010) The orphan tyrosine kinase receptor, ROR2, mediates Wnt5A signaling in metastatic melanoma. Oncogene 29: 34–44. 10.1038/onc.2009.305 19802008 PMC2803338

[83] Saha S, Bardelli A, Buckhaults P, Velculescu VE, Rago C, St Croix B, et al (2001) A phosphatase associated with metastasis of colorectal cancer. Science 294: 1343–1346. 11598267 10.1126/science.1065817

[84] Wright TM, Brannon AR, Gordan JD, Mikels AJ, Mitchell C, Chen S, et al (2009) Ror2, a developmentally regulated kinase, promotes tumor growth potential in renal cell carcinoma. Oncogene 28: 2513–2523. 10.1038/onc.2009.116 19448672 PMC2771692

[85] Ataseven B, Gunesch A, Eiermann W, Kates RE, Hogel B, Knyazev P, et al (2014) PTK7 as a potential prognostic and predictive marker of response to adjuvant chemotherapy in breast cancer patients, and resistance to anthracycline drugs. OncoTargets and therapy 7: 1723–1731. 10.2147/OTT.S62676 25336969 PMC4199823

[86] Enomoto M, Hayakawa S, Itsukushima S, Ren DY, Matsuo M, Tamada K, et al (2009) Autonomous regulation of osteosarcoma cell invasiveness by Wnt5a/Ror2 signaling. Oncogene 28: 3197–3208. 10.1038/onc.2009.175 19561643

[87] Kobayashi M, Shibuya Y, Takeuchi J, Murata M, Suzuki H, Yokoo S, et al (2009) Ror2 expression in squamous cell carcinoma and epithelial dysplasia of the oral cavity. Oral Surg Oral Med Oral Pathol Oral Radiol Endod 107: 398–406. 10.1016/j.tripleo.2008.08.018 19217015

[88] Morioka K, Tanikawa C, Ochi K, Daigo Y, Katagiri T, Kawano H, et al (2009) Orphan receptor tyrosine kinase ROR2 as a potential therapeutic target for osteosarcoma. Cancer Sci 100: 1227–1233. 10.1111/j.1349-7006.2009.01165.x 19486338 PMC11158182

[89] Lara E, Calvanese V, Huidobro C, Fernandez AF, Moncada-Pazos A, Obaya AJ, et al (2010) Epigenetic repression of ROR2 has a Wnt-mediated, pro-tumourigenic role in colon cancer. Mol Cancer 9: 170 10.1186/1476-4598-9-170 20591152 PMC2903502

[90] Prebet T, Lhoumeau AC, Arnoulet C, Aulas A, Marchetto S, Audebert S, et al (2010) The cell polarity PTK7 receptor acts as a modulator of the chemotherapeutic response in acute myeloid leukaemia and impairs clinical outcome. Blood. 10.1182/blood-2010-01-262352 20558616

[91] Gartner S, Gunesch A, Knyazeva T, Wolf P, Hogel B, Eiermann W, et al (2014) PTK 7 is a transforming gene and prognostic marker for breast cancer and nodal metastasis involvement. PloS one 9: e84472 10.1371/journal.pone.0084472 24409301 PMC3883666

[92] Liu Q, Zhang C, Yuan J, Fu J, Wu M, Su J, et al (2014) PTK7 regulates Id1 expression in CD44-high glioma cells. Neuro-oncology. 10.1093/neuonc/nou227 PMC4483067 25204555

[93] Zhang H, Wang A, Qi S, Cheng S, Yao B, Xu Y (2014) Protein tyrosine kinase 7 (PTK7) as a predictor of lymph node metastases and a novel prognostic biomarker in patients with prostate cancer. International journal of molecular sciences 15: 11665–11677. 10.3390/ijms150711665 24987951 PMC4139806

[94] Golubkov VS, Chekanov AV, Cieplak P, Aleshin AE, Chernov AV, Zhu W, et al (2010) The Wnt/planar cell polarity (PCP) protein tyrosine kinase-7 (PTK7) is a highly efficient proteolytic target of membrane type-1 matrix metalloproteinase (MT1-MMP): implications in cancer and embryogenesis. J Biol Chem. 10.1074/jbc.M110.165159 PMC2975198 20837484

[95] Carter RE, Sorkin A (1998) Endocytosis of functional epidermal growth factor receptor-green fluorescent protein chimera. The Journal of biological chemistry 273: 35000–35007. 9857032 10.1074/jbc.273.52.35000

