# Supplementary figures and images for: A PTK7/Ror2 Co-Receptor Complex Affects Xenopus Neural Crest Migration
Source: PLoS One. 2015 Dec 17;10(12):e0145169. doi: 10.1371/journal.pone.0145169 (PMC4683079; doi:10.1371/journal.pone.0145169)

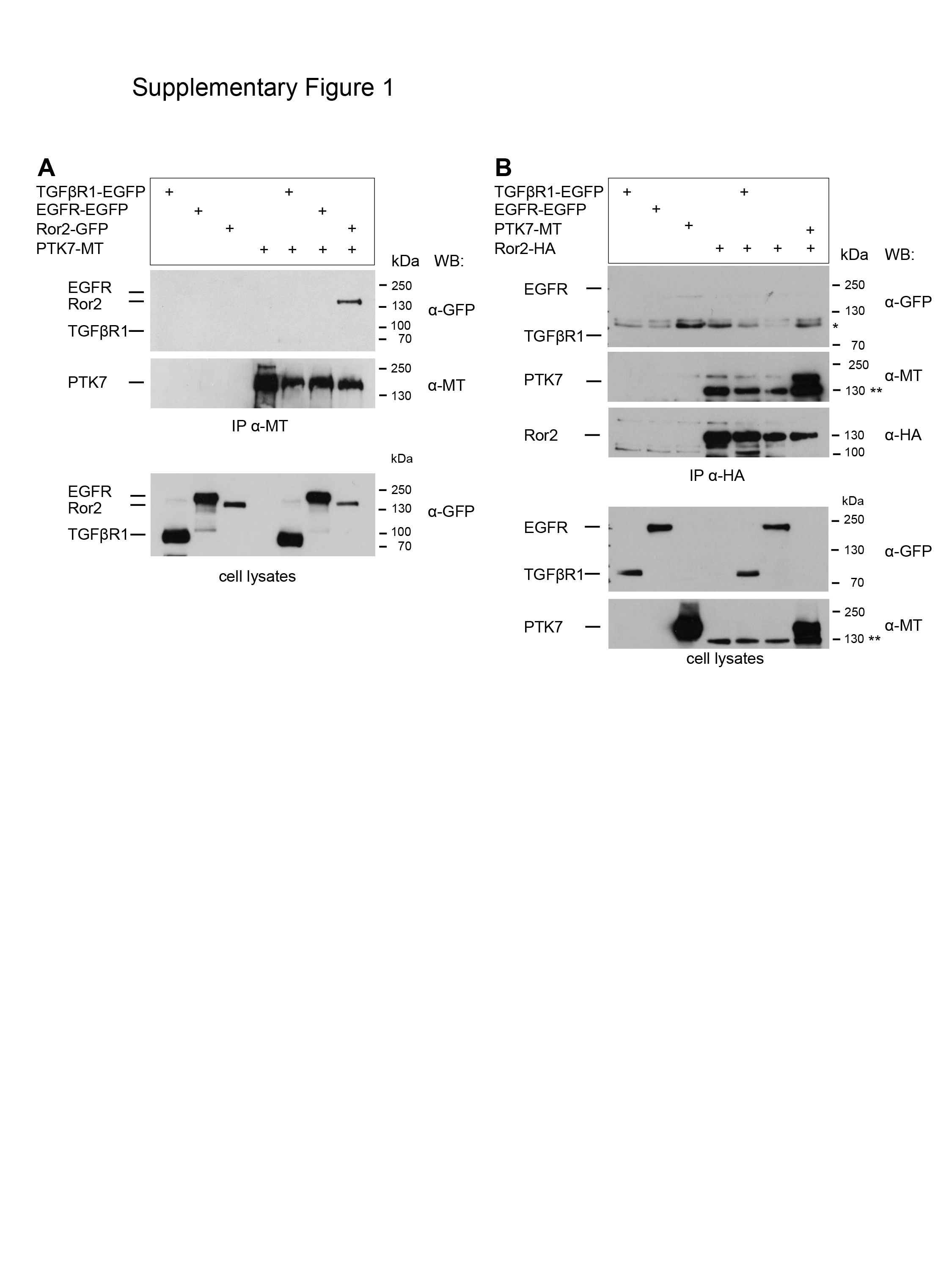

Supplement: S1 Fig — A Full-length myc-tagged PTK7 (PTK7-MT) was co-expressed with Ror2-EGFP, EGFR-EGFP [95] or TGFß1R-EGFP (kind gift of A. Menke, Molecular Oncology of Solid Tumors, Giessen, Germany) as indicated in MCF7 cells. Cell lysates were precipitated using anti-myc antibodies (IP α-MT, upper panel). Precipitates are shown in the upper panels, cell lysates in the lower panel. Antibodies used for Western blotting and molecular weights are indicated at the right. B Full-length HA-tagged Ror2 was co-expressed with PTK7-MT, EGFR-EGFP or TGFß1R-GFP as indicated in MCF7 cells and cell lysates were precipitated using anti-HA antibodies (IP α-HA, upper panel). Precipitates are shown in the three upper panels and lysates in the two lower panels. Antibodies used for Western blotting and molecular weights are indicated at the right; * marks unspecific bands, ** Ror2 signal remaining from previous anti-HA staining, which was only partially removed by blot stripping. (TIF) [file pone.0145169.s001.tif]

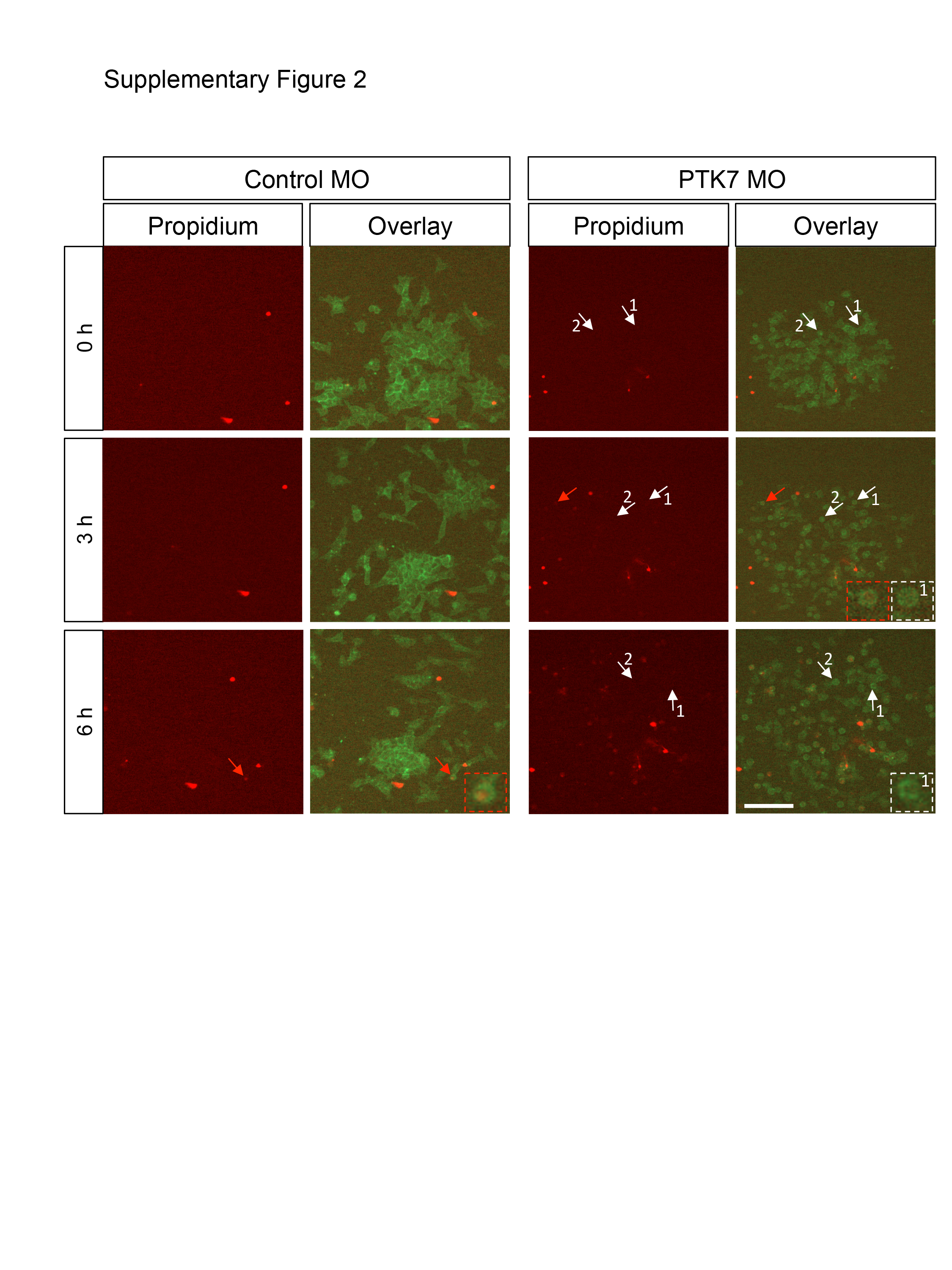

Supplement: S2 Fig — NC explants injected with 7.5 ng control MO or PTK7 MO in combination with 50 pg mGFP were treated with PI (10 μg/ml) to test for the viability of the explanted NC cells. Cell membrane integrity prevents PI staining of viable cells, while it can stain nucleic acids of apoptotic cells (red). In PTK7 morphant cells few PI-positive cells appear after 3 hours compared to 6 hours in controls (one example each is marked by a red arrow). Round-shaped PTK7 morphant NC cells appear early in the experiment and keep moving/blebbing for up to a couple of hours (white arrows, numbers indicate specific cells during the course of the experiment). Thus, the roundish cell shape is not necessarily an indication of cell death. Dashed squares show higher magnifications of specific cells. Scale bar = 50 μm. (TIF) [file pone.0145169.s002.tif]
